# Odontogenic keratocyst: imaging features of a benign lesion with an aggressive behaviour

**DOI:** 10.1007/s13244-018-0644-z

**Published:** 2018-07-31

**Authors:** Andrea Borghesi, Cosimo Nardi, Caterina Giannitto, Andrea Tironi, Roberto Maroldi, Francesco Di Bartolomeo, Lorenzo Preda

**Affiliations:** 1 Department of Radiology, University of Brescia, Brescia, Italy; 2 Spedali Civili di Brescia, Brescia, Italy; 3 Department of Experimental and Clinical Biomedical Sciences, Radiodiagnostic Unit Number 2, University of Florence, Florence, Italy; 4 Azienda Ospedaliera Universitaria Careggi, Florence, Italy; 5 Division of Radiology, European Institute of Oncology, Milan, Italy; 6 Department of Pathology, Spedali Civili di Brescia, Brescia, Italy; 7 Postgraduate School in Radiodiagnostics, Università degli Studi di Milano, Milan, Italy; 8 Department of Clinical-Surgical, Diagnostic and Pediatric Sciences, University of Pavia, Pavia, Italy; 9 Diagnostic Imaging Unit, National Centre of Oncological Hadrontherapy (CNAO), Pavia, Italy

**Keywords:** Odontogenic keratocysts, Panoramic radiography, Computed tomography, Magnetic resonance imaging, Basal cell nevus syndrome

## Abstract

**Abstract:**

The latest (4th) edition of the World Health Organization (WHO) Classification of Head and Neck Tumours, published in January 2017, has reclassified keratocystic odontogenic tumour as odontogenic keratocyst. Therefore, odontogenic keratocysts (OKCs) are now considered benign cysts of odontogenic origin that account for about 10% of all odontogenic cysts. OKCs arise from the dental lamina and are characterised by a cystic space containing desquamated keratin with a uniform lining of parakeratinised squamous epithelium. The reported age distribution of OKCs is considerably wide, with a peak of incidence in the third decade of life and a slight male predominance. OKCs originate in tooth-bearing regions and the mandible is more often affected than the maxilla. In the mandible, the most common location is the posterior sextant, the angle or the ramus. Conversely, the anterior sextant and the third molar region are the most common sites of origin in the maxilla. OKCs are characterised by an aggressive behaviour with a relatively high recurrence rate, particularly when OKCs are associated with syndromes. Multiple OKCs are typically associated with the nevoid basal cell carcinoma syndrome (NBCCS), an autosomal dominant multisystemic disease. Radiological imaging, mainly computed tomography (CT) and, in selected cases, magnetic resonance imaging (MRI), plays an important role in the diagnosis and management of OKCs. Therefore, the main purpose of this pictorial review is to present the imaging appearance of OKCs underlining the specific findings of different imaging modalities and to provide key radiologic features helping the differential diagnoses from other cystic and neoplastic lesions of odontogenic origin.

**Key Points:**

• *Panoramic radiography is helpful in the preliminary assessment of OKCs*.

• *CT is considered the tool of choice in the evaluation of OKCs*.

• *MRI with DWI or DKI can help differentiate OKCs from other odontogenic lesions*.

• *Ameloblastoma, dentigerous and radicular cysts should be considered in the differential diagnosis*.

• *The presence of multiple OKCs is one of the major criteria for the diagnosis of NBCCS*.

## Introduction

Odontogenic keratocysts (OKCs), first described by Philipsen in 1956 [1], are benign intraosseous lesions of odontogenic origin that account for about 10% of jaw cysts. They are characterised by an aggressive behaviour with a relatively high recurrence rate [2]. Histologically, OKCs arise from the dental lamina and are constituted by a cystic space containing desquamated keratin, lined with a uniform parakeratinised squamous epithelium of 5 to 10 cell layers, with a distinct basal layer of palisaded columnar or cuboidal cells, whose nuclei tend to be vertically oriented. The interface with the adjacent connective tissue is normally flat with a potential for budding of the basal layer and the formation of small satellite cysts [3]. The mitotic activity is higher than other cysts of odontogenic origin [4].

Because of this histologic feature, the aggressive behaviour and the fact that a large proportion of lesions are associated with a mutation or inactivation of the tumour suppressor gene, also called the protein patched homolog (PTCH) gene, in the 3rd edition of the World Health Organization (WHO) Classification of Head and Neck Tumours, this pathological entity was included in the group of odontogenic neoplasms with the name of keratocystic odontogenic tumour (KCOT) [5].

In the latest (4th) edition of the WHO Classification of Head and Neck Tumours published in January 2017 [6], the consensus group concluded that, at the present time, there is insufficient evidence to support a neoplastic origin of this cystic lesion and that further research is needed [7]. Consequently, the name OKC has been reinserted, replacing the term KCOT that was removed from the classification.

Preoperative assessment is important for planning treatment and management, as OKCs require a more aggressive treatment than other low-attenuating lesions having similar radiological appearance.

The aim of this pictorial review is to present the imaging appearance of OKCs underlining the specific findings of different imaging modalities and to provide key radiologic features helping the differential diagnoses from other cystic and neoplastic lesions of odontogenic origin.

## Incidence, clinical presentation and natural history

OKCs represent approximately 10% of odontogenic cysts and the reported age distribution is considerably wide (from 8 to 82 years), with a peak of incidence in the third decade of life [3, 8, 9]. Most series have shown a slight preponderance in males [10].

The presence of multiple OKCs, also occurring in different moments during the lifetime of the patients, is typically associated with the nevoid basal cell carcinoma syndrome (NBCCS), also known as Gorlin–Goltz syndrome, an autosomal dominant multisystemic disease. In these patients, the mean age of incidence decreases to about 25 years old [11–13].

Similarly to other entities having an odontogenic origin, OKCs originate in tooth-bearing regions. They occur twice as often in the mandible as in the maxilla [14]. When OKCs originate from the mandible, the most common location is the posterior sextant, the angle or the ramus [15, 16]. Conversely, the anterior sextant, mainly between canine and lateral incisor, and the third molar region are the most common sites of origin in the maxilla [17, 18]. Large size lesions are particularly common at the angle and ramus of the mandible [19]. According to the literature, OKCs may be located in a periapical position, in a pericoronal position or in a lateral root position. In about 30% of cases, they have no relationships with any dental structures [10, 17]. In spite of their aggressive behaviour, OKCs, in most cases, cause minimal bone expansion because of their propensity to spread along the intramedullary space “growing in the length of the bone” [20]. Large lesions, causing significant erosion of cortical plates and involvement of surrounding structures, may be seen in asymptomatic patients [21]. Consequently, especially in western countries, the presence of OKCs may be found at a later stage as an incidental finding during routine radiological investigations. A systematic review of the literature published in 2011 by MacDonald-Jankowski showed that patients of East Asian origin may present symptoms early, characterised by swelling and pain, while discharge and numbness of the inferior alveolar nerve are described more frequently in Latin Americans [22]. Unlike other odontogenic lesions having similar aggressive behaviour such as ameloblastomas, OKCs infrequently cause root resorption of adjacent teeth [10].

The reported recurrent rate of OKCs after surgery is wide, up to 30%, with most recurrences occurring after conservative treatments of simple lesion’s enucleation [2, 19, 23].

Higher recurrence rates are reported in patients affected by NBCCS and in multilocular lesions [24, 25]. The recurrences might be explained by different causes: incomplete removal of highly active basal layer of the epithelial cyst lining, growth of small intramedullary satellite cysts left behind by conservative treatment and development of lesions localised in the adjacent region of the jaws [13, 19, 26]. The type of surgery may not be the only factor and some authors suggested that recurrence may be related with the biological nature of the lesion itself and the expression of proliferative markers such as Ki-67 [27, 28].

## Imaging techniques

The radiological imaging techniques most commonly used in the study of OKCs are conventional radiography (mainly panoramic radiography), computed tomography (CT) and magnetic resonance imaging (MRI). These imaging modalities differ significantly in their technical characteristics, acquisition modalities, indications and information provided.

### Panoramic radiography

Panoramic radiography is a flat representation of the curved surfaces of the maxillary and mandibular dental arches and is helpful in the preliminary assessment of the location, size, shape, margins and extension of odontogenic lesions, such as OKCs. However, this radiographic technique has a limited role because it provides a two-dimensional view of maxillofacial structures with magnification, geometric distortion and overlapping. Therefore, to overcome the limitations of panoramic radiography, a three-dimensional imaging modality is often required for preoperative planning, particularly in larger lesions.

**Fig. 1:**
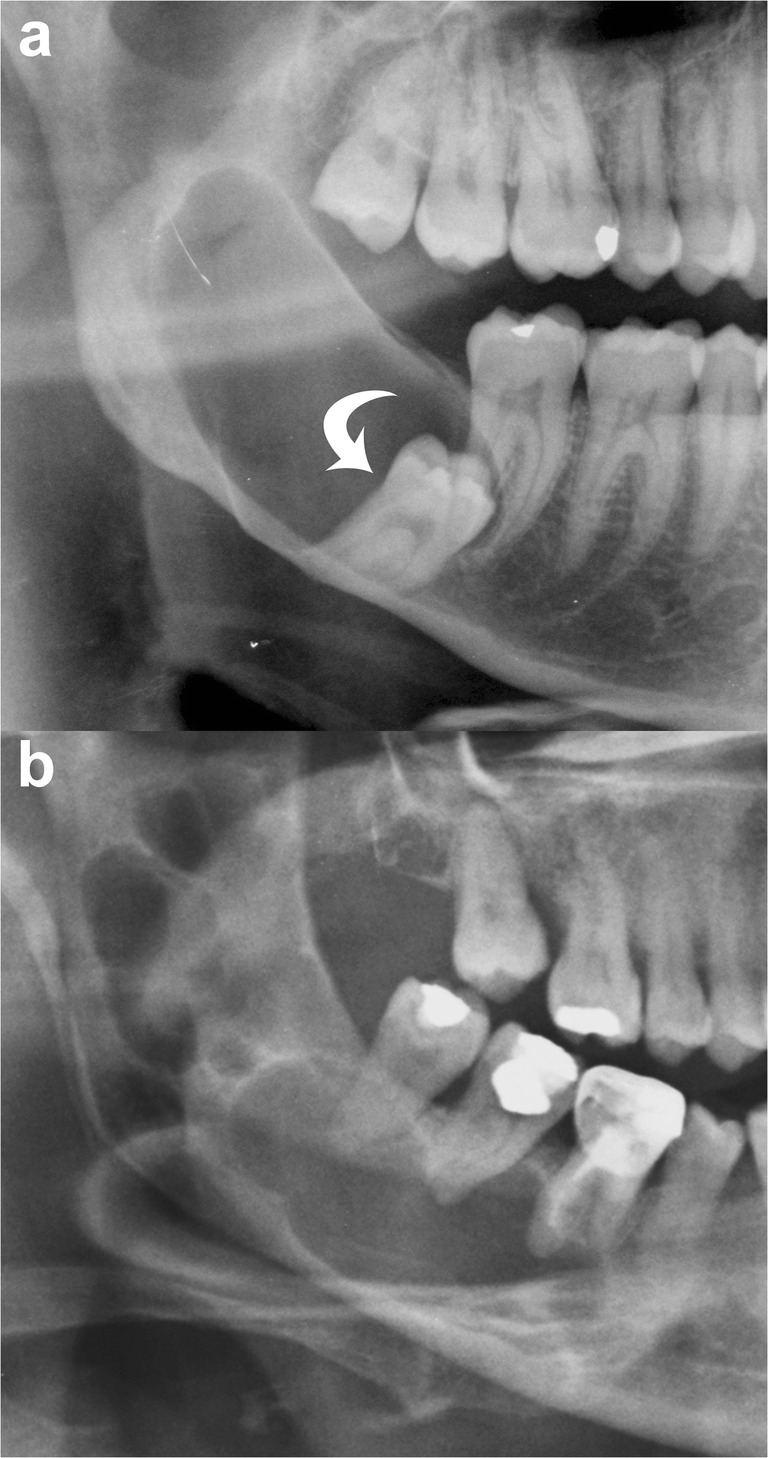
Mandibular odontogenic keratocysts (OKCs). **a** Cropped panoramic radiograph shows a unilocular lesion in the posterior mandible and ramus that determines mesial displacement of the impacted third molar (*curved arrow*). **b** Cropped panoramic radiograph demonstrates a multilocular lesion occupying the posterior mandible and ramus with a soap-bubble pattern

**Fig. 2:**
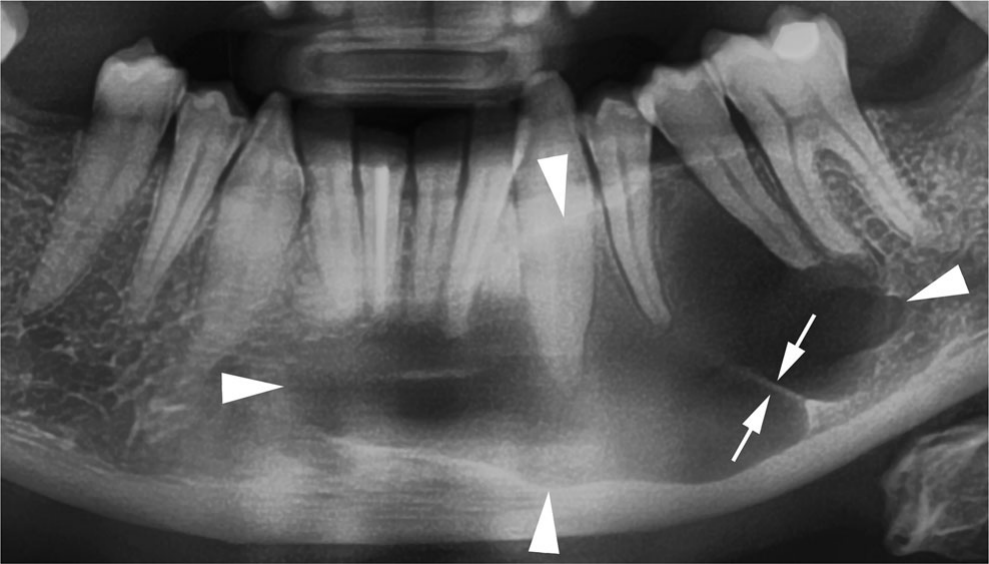
Cropped panoramic radiograph shows a large OKC with well-defined and lobulated margins (*arrowheads*) occupying the body of the mandible. Note an incomplete internal septum within the lesion (*opposing arrows*)

Radiographically, OKCs appear as a well-defined unilocular or multilocular radiolucency bounded by corticated margins (Fig. 1). Unilocular lesions are predominant, whereas the multilocular variant is observed in approximately 30% of cases, most commonly in the mandible (Fig. 1b) [9, 29]. On panoramic radiography, mandibular unilocular OKCs may show few and incomplete septa within the lesions; this finding is more common in larger than in smaller OKCs (Fig. 2).

Approximately 30% of OKCs are associated with at least one unerupted tooth, most commonly the third molars (Fig. 1a) [9, 29]. This association occurs particularly in younger patients [15].

**Fig. 3:**
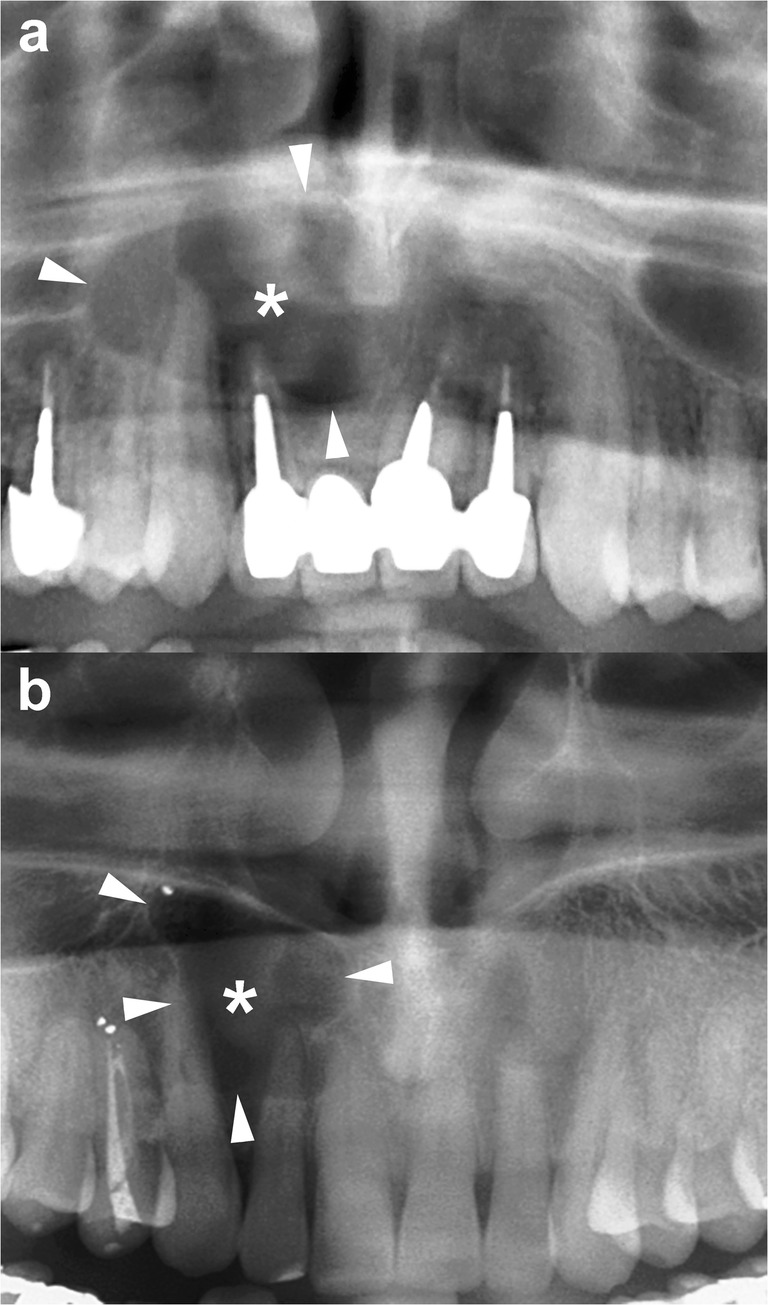
Histologically proven OKCs. Cropped panoramic radiographs (**a** and **b**) show two unilocular radiolucent lesions (*asterisks*) with well-defined and corticated margins (*arrowheads*) located in the anterior maxilla, between the roots of the adjacent teeth. The radiographic aspect of these radiolucent lesions may simulate a radicular cyst

The radiographic features of OKCs are not pathognomonic, particularly in smaller unilocular lesions [15]. When a small unilocular OKC occurs in the anterior sextant of the maxilla, it may simulate other odontogenic and non-odontogenic cysts, such as radicular cyst (Fig. 3), lateral periodontal cyst or nasopalatine cyst [17, 30].

**Fig. 4:**
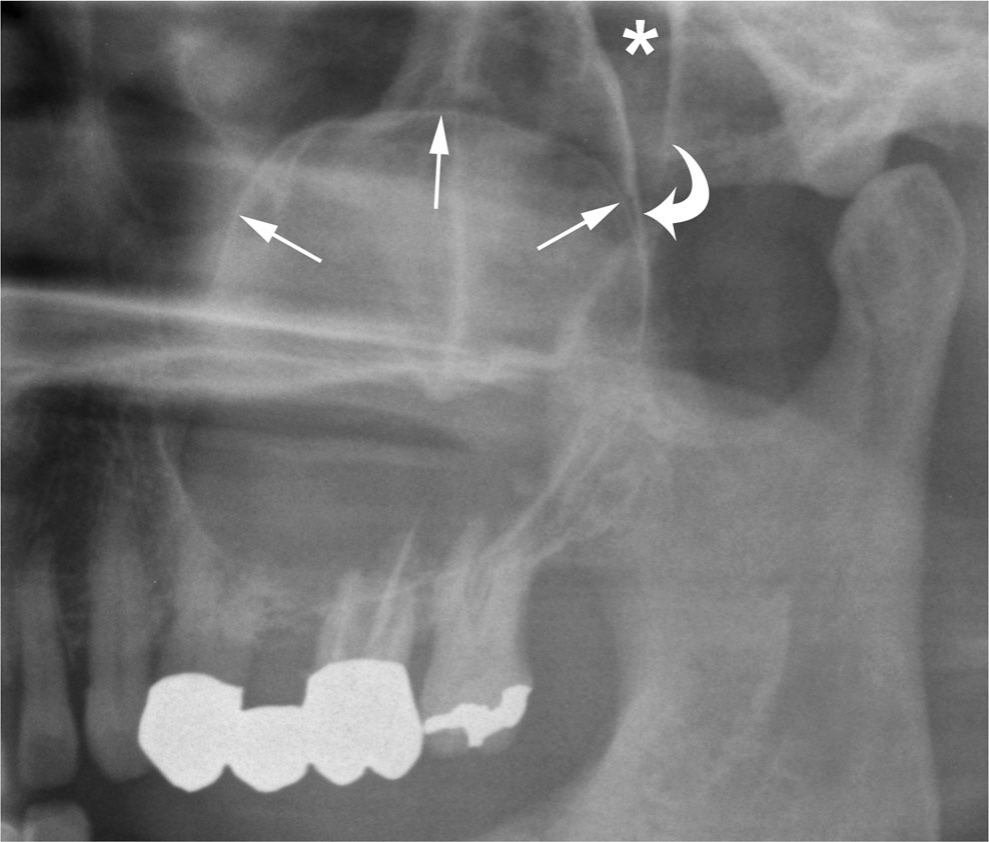
Cropped panoramic radiograph of the posterior left maxilla demonstrates a large OKC extending into the maxillary sinus (*arrows*). The posterior wall of the maxillary sinus (*curved arrow*) and the pterygopalatine fossa (*asterisk*) are also displayed

Large mandibular OKCs tend to grow predominantly along the length of the bone with minimal bucco-lingual expansion, especially within the body [15]. On panoramic radiography, this peculiar pattern of growth may determine an extensive radiolucent lesion with considerable mesiodistal dimensions and without a significant cortical expansion (Figs. 1a and 2). On the other hand, large maxillary OKCs display a significant expansion of the alveolar bone and tend to involve adjacent structures. In particular, when a maxillary OKC originate from the molar region, an extension into the maxillary sinus is frequently seen (Fig. 4) [22].

Radiographically, OKCs may show tooth displacement and root resorption; this latter finding is an uncommon radiographic feature of OKCs, with a reported incidence varying from 1.3 to 11% [9]. The literature reported that the perforation of the cortical bone is not an unusual feature of OKCs, with an intraoperative incidence varying from 39 to 51% [9]. However this finding is detected very rarely on panoramic radiography and is generally limited to the alveolar crest.

### Cone beam and multidetector computed tomography

In clinical routine, there are two main CT techniques commonly used for the evaluation of maxillofacial diseases: cone beam CT (CBCT) and multidetector CT (MDCT). Both CT modalities are usually considered adequate for diagnosing OKCs and preoperative planning, owing to their ability to generate high-quality multiplanar reconstruction (MPR) images in different planes. In addition, using a dedicated reconstruction software for dental arches (DentaScan), the three-dimensional dataset produced by both modalities can be further processed in MPR images that are either parallel (panoramic) or perpendicular (cross-sectional) to the curvature of the alveolar bones. These high-resolution MPR images allow three-dimensional views of the jaws and provide detailed information about the OKC and its relationship with surrounding structures (teeth, sinonasal cavities, canals, foramina and soft tissue).

The main advantage that makes CBCT a particularly attractive technique in the evaluation of maxillary and mandibular lesions is its higher spatial resolution compared with MDCT. Conversely, the main disadvantage of CBCT is the poor contrast resolution, which is not suitable for soft tissue contrast discrimination. Hence, CBCT is not able to evaluate the extension into soft tissues and precludes the possibility of contrast medium injection [31]. In the assessment of an OKC, CBCT is considered more effective to demonstrate the bony changes of the cortical plates of jaws (buccal, palatal or lingual cortices), whereas MDCT is more effective at evaluating internal density and extension into soft tissue.

**Fig. 5:**
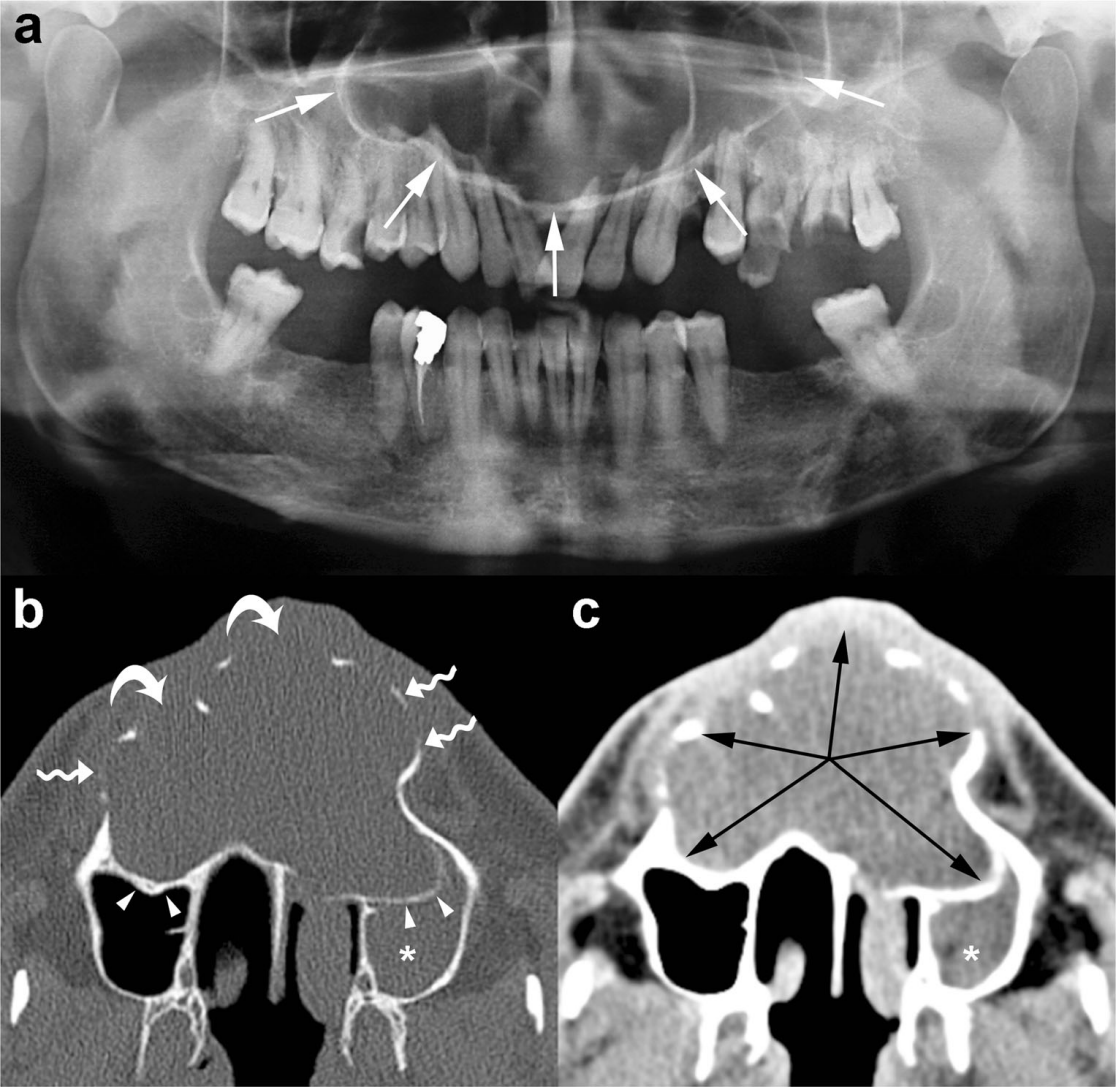
Maxillary OKC. Panoramic radiograph (**a**) shows a large radiolucency with a well-defined and corticated rim in the maxilla (*white arrows*). Axial multidetector computed tomography (MDCT) images with bone window (**b**) and soft tissue window (**c**) clearly demonstrate the hydraulic expansion of the maxillary alveolar bone (*black arrows*) with thinning (*wavy arrows*) and perforation (*curved arrows*) of the buccal cortex. Posterior bowing of the floor of the maxillary sinuses (*arrowheads*) and inflammatory material within the left maxillary sinus (*asterisks*) are also shown

As with panoramic radiography, CT is able to display the main radiological features of an OKC, such as size, shape (hydraulic or scalloping), margins (well-defined and corticated), internal appearance (uni- or multilocular) and effects on adjacent structures (tooth displacement, root resorption, maxillary sinus floor elevation, inferior displacement of mandibular canal) [32]. In addition, CT demonstrates other features of OKCs, such as bony changes (expansion in buccolingual/palatal direction and erosion), internal density and extension into soft tissue (Fig. 5).

Therefore, CT is considered superior to conventional radiography in differentiating OKCs from other unilocular or multilocular osteolytic lesions and in the preoperative assessment (Fig. 5).

**Fig. 6:**
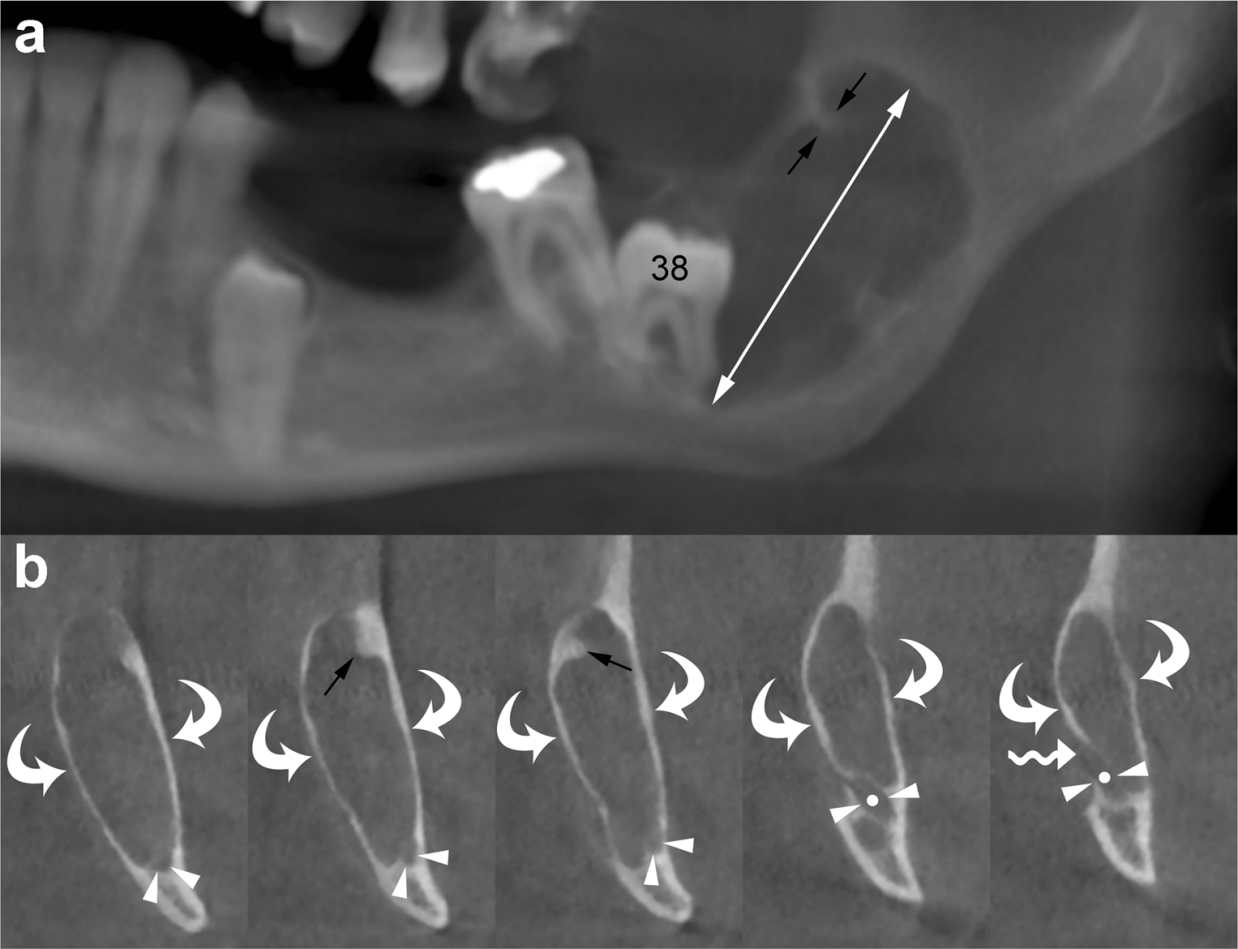
Mandibular OKC. Panoramic (**a**) and cross-sectional (**b**) cone beam computed tomography (CBCT) images display an osteolytic odontogenic lesion in the posterior left mandible and ramus, with a growth predominantly along the length of the bone (*double-headed arrow*) and minimal expansion of the buccal and lingual cortices (*curved arrows*). Note mesial displacement of the impacted third molar (*38*) and inferior displacement of the mandibular canal (*arrowheads and dots*). Small and incomplete internal septum (*small black arrows*) due to the endosteal scalloping of the cortical plate are also shown. *Wavy arrow*, mandibular foramen

**Fig. 7:**
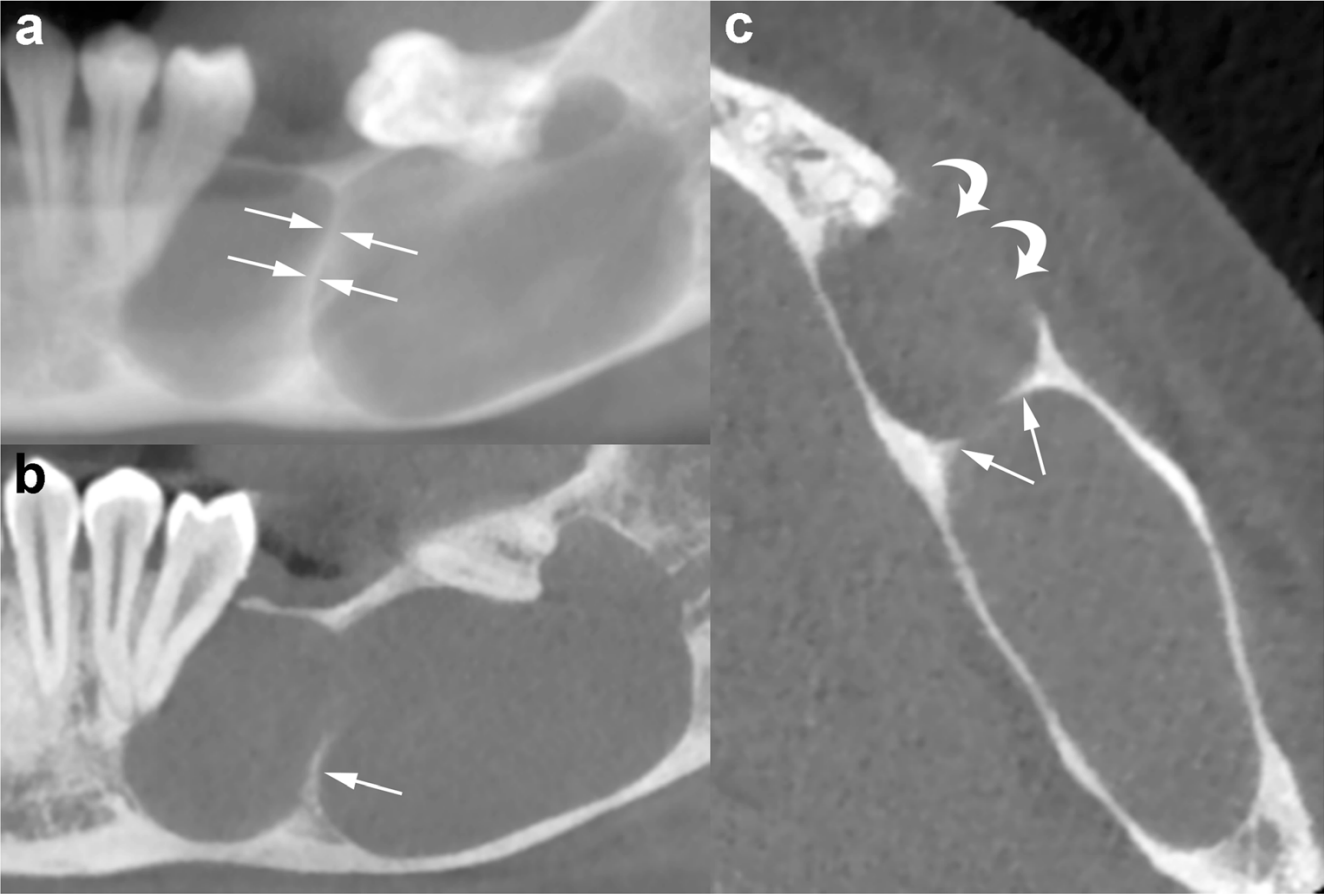
Panoramic CBCT image with 20-mm slice thickness (**a**) shows a mandibular OKC with a septum (*opposing arrows*) which seems to divide the lesion into two large loculations. Note the displacement of adjacent teeth. Panoramic (**b**) and axial (**c**) CBCT images reconstructed as 0.5- and 0.2-mm-thick sections demonstrate that the septum is incomplete (*arrows*). Perforation of the buccal cortex in the anterior portion of the lesion is also shown (*curved arrows*)

**Fig. 8:**
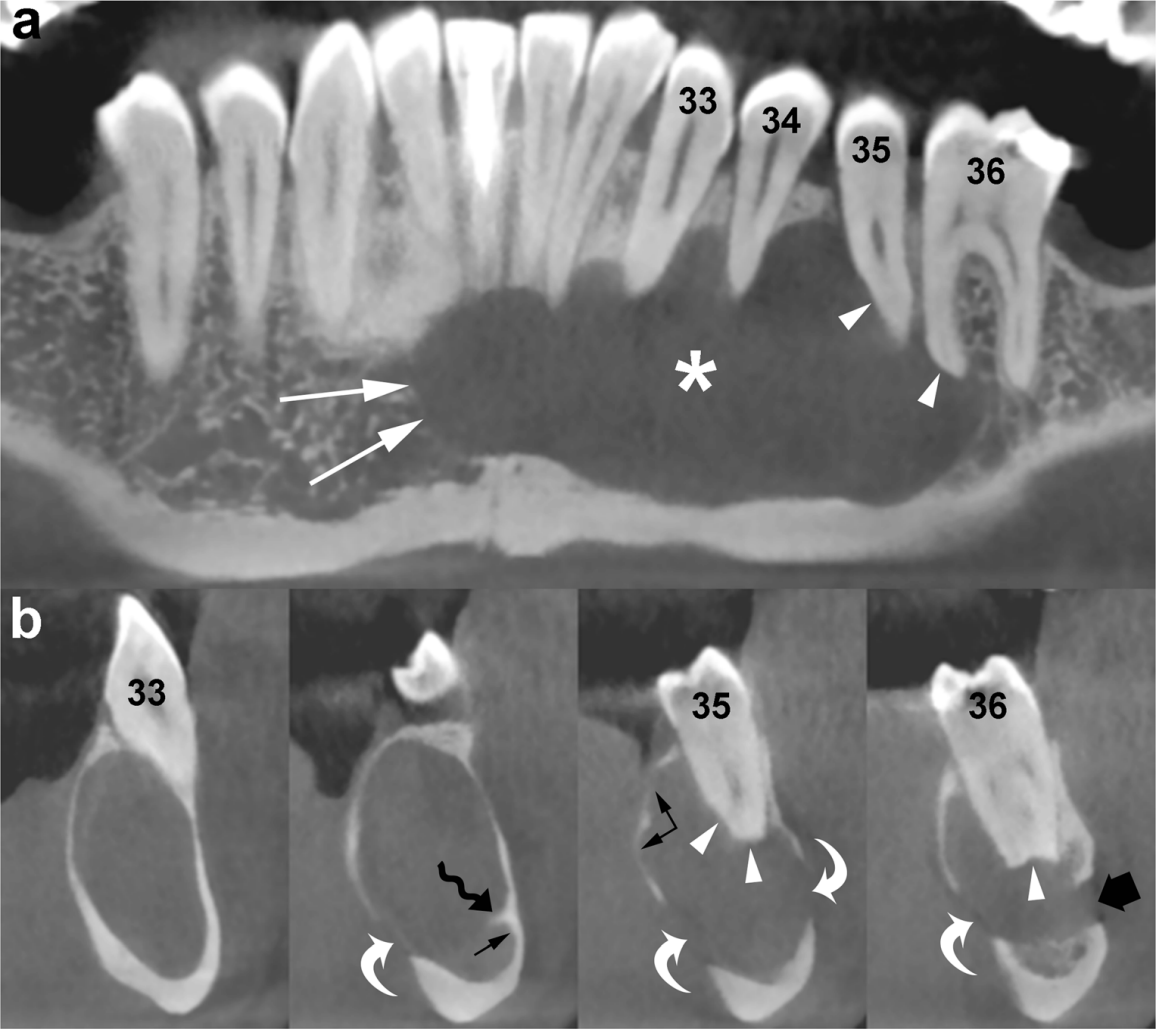
Panoramic (**a**) and cross-sectional (**b**) CBCT images show an OKC with well-defined and lobulated margins located in the interforaminal region of the mandible (*asterisk*). The lesion grows mesially by crossing the midline (*white arrows*). Note root resorption (*arrowheads*) and perforation of the cortices (*curved arrows*). Scalloping of the endosteal surface of the cortical plates (*small black arrows*) and small internal septum (*wavy arrow*) are also seen. *Large black arrow*, left mental foramen; *33*, left canine; *34*, left first premolar; *35*, left second premolar; *36*, left first molar

In the mandible, the OKCs have a tendency to grow predominantly mesiodistally along the length of the bone, causing minimal expansion of the buccal and lingual cortical plates (Fig. 6) [33]. However, in some cases, the OKC may expand and erode the cortices (Figs. 7 and 8).

**Fig. 9:**
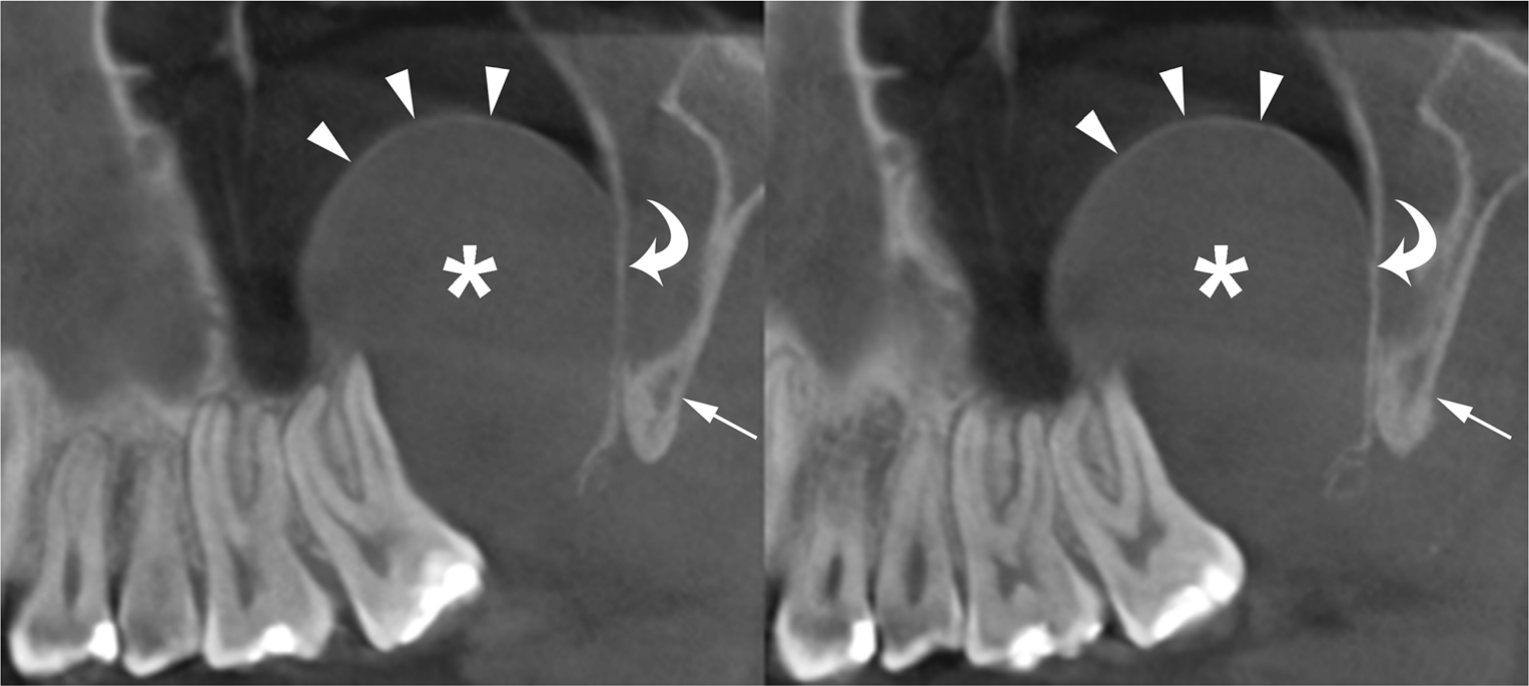
Panoramic CBCT images of a maxillary OKC (*asterisks*) originating from the molar region distally to the second molar tooth. The OKC causes significant sinus floor elevation (*arrowheads*). *Curved arrows*, posterior wall of the maxillary sinus; *arrows*, lateral pterygoid lamina

In contrast, large OKCs in the maxilla more frequently present a hydraulic expansion of the alveolar bone with remodelling, thinning, scalloping and perforation of the cortices (Fig. 5) [32]. In addition, when OKCs originate from the alveolar bone subjacent to the maxillary sinus, its floor is lifted and lumen is reduced (Fig. 9).

**Fig. 10:**
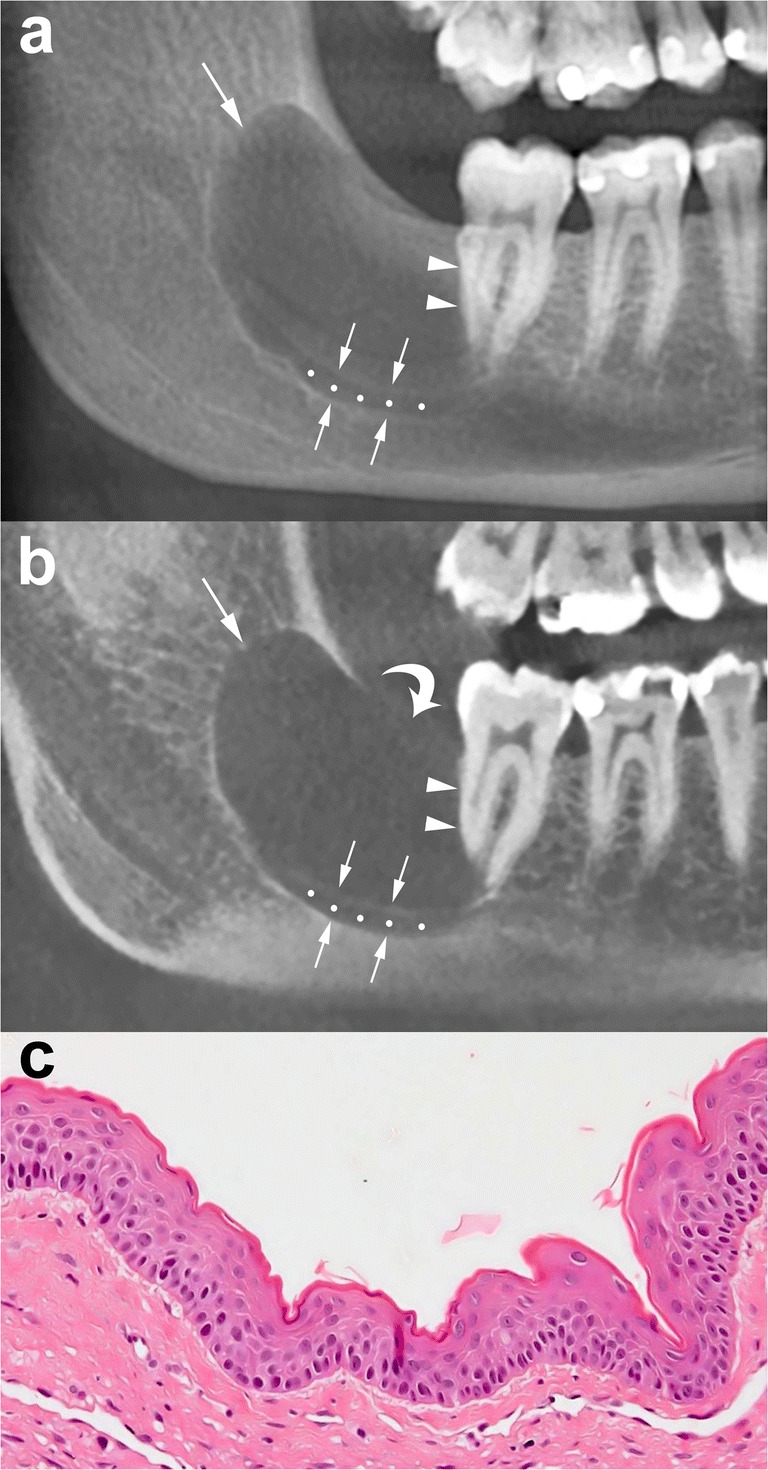
Panoramic CBCT images with 20-mm (**a**) and 0.5-mm (**b**) slice thickness of an OKC show a unilocular lesion with well-defined and corticated margins located in the posterior sextant and ramus of the right mandible (*arrows*), near the distal root of the second molar (*arrowheads*). Note the interruption of the superior border of the retromolar region (*curved arrow*) and inferior displacement of the mandibular canal (*opposing arrows and dots*). **c** Histological image shows the typical parakeratinised stratified squamous epithelial lining with corrugated surfaces (H-E 10×)

**Fig. 11:**
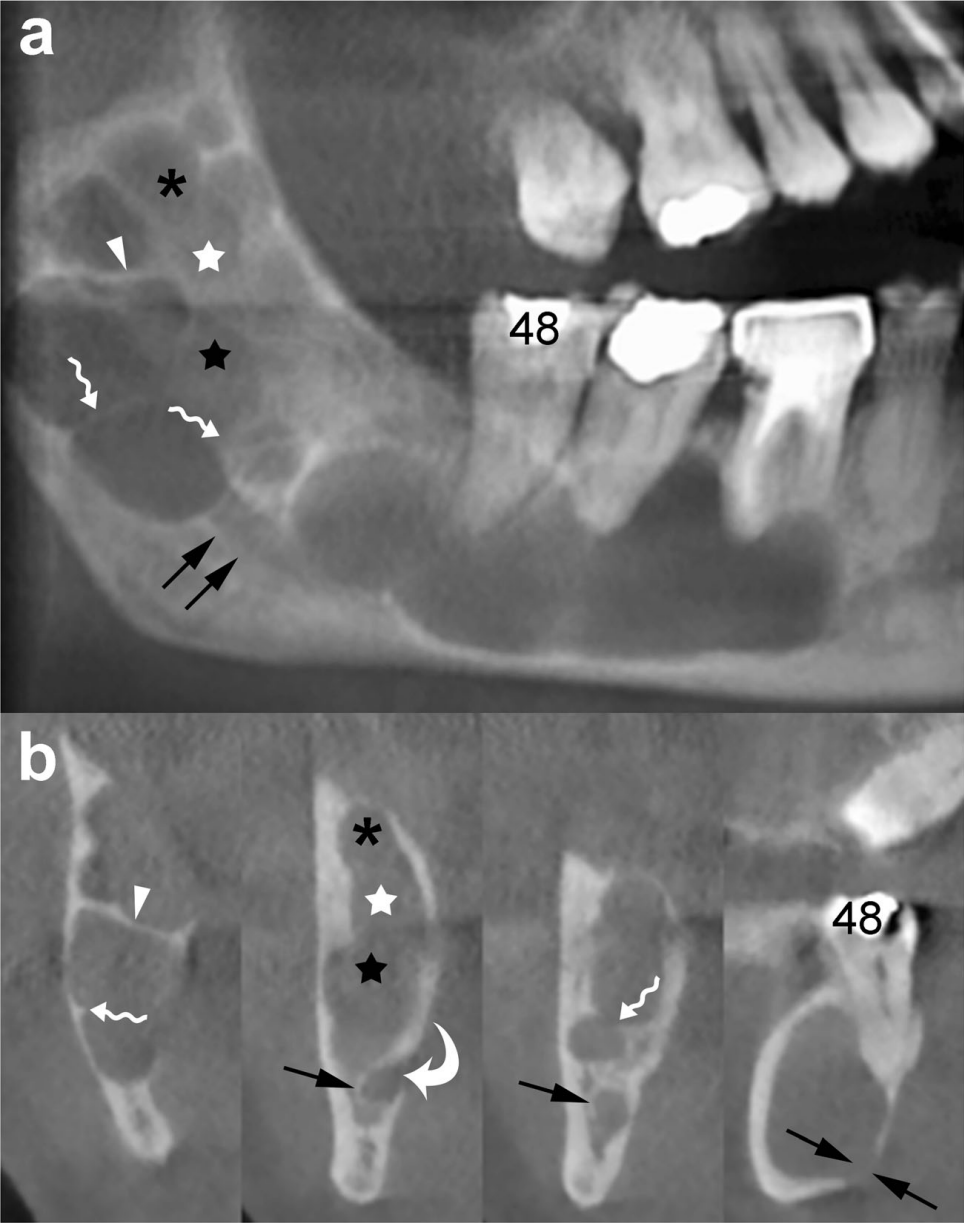
Panoramic CBCT image with 20-mm slice thickness (**a**) shows a multilocular OKC. On this reconstruction, septa seem to divide the lesion into multiple loculations. Cross-sectional CBCT images reconstructed as 0.5-mm-thick sections (**b**) demonstrate that some of these septa are complete (*arrowheads*) and some are incomplete (*wavy arrows*). Note that certain small loculations shown by the panoramic CBCT image (*black asterisk*, *white and black stars*) actually correspond to a single large loculation with scalloped borders. Compression and lingual displacement of the mandibular canal, deep to the root of the third molar (*48*), is also shown (*opposing arrows*). *Curved arrow*, mandibular foramen; *arrows*, mandibular canal

The difference between the growth pattern of mandibular and maxillary OKCs may be partly due to the higher cortical thickness of the mandible compared to that of the maxilla [15]. On CT images, OKCs typically manifest as osteolytic lesions that exhibit a unilocular (Figs. 9 and 10) or a predominantly unilocular morphology with few and incomplete septa (Figs. 7 and 8). The multilocular presentation with adjacent satellite cysts (daughter cysts) is possible, particularly in large lesions (Fig. 11). In these cases, loculations are usually large and few (soap-bubble appearance).

OKCs may be associate with an impact tooth (Fig. 6); this finding, similar to dentigerous cyst, is more common in younger patients [14, 15].

**Fig. 12:**
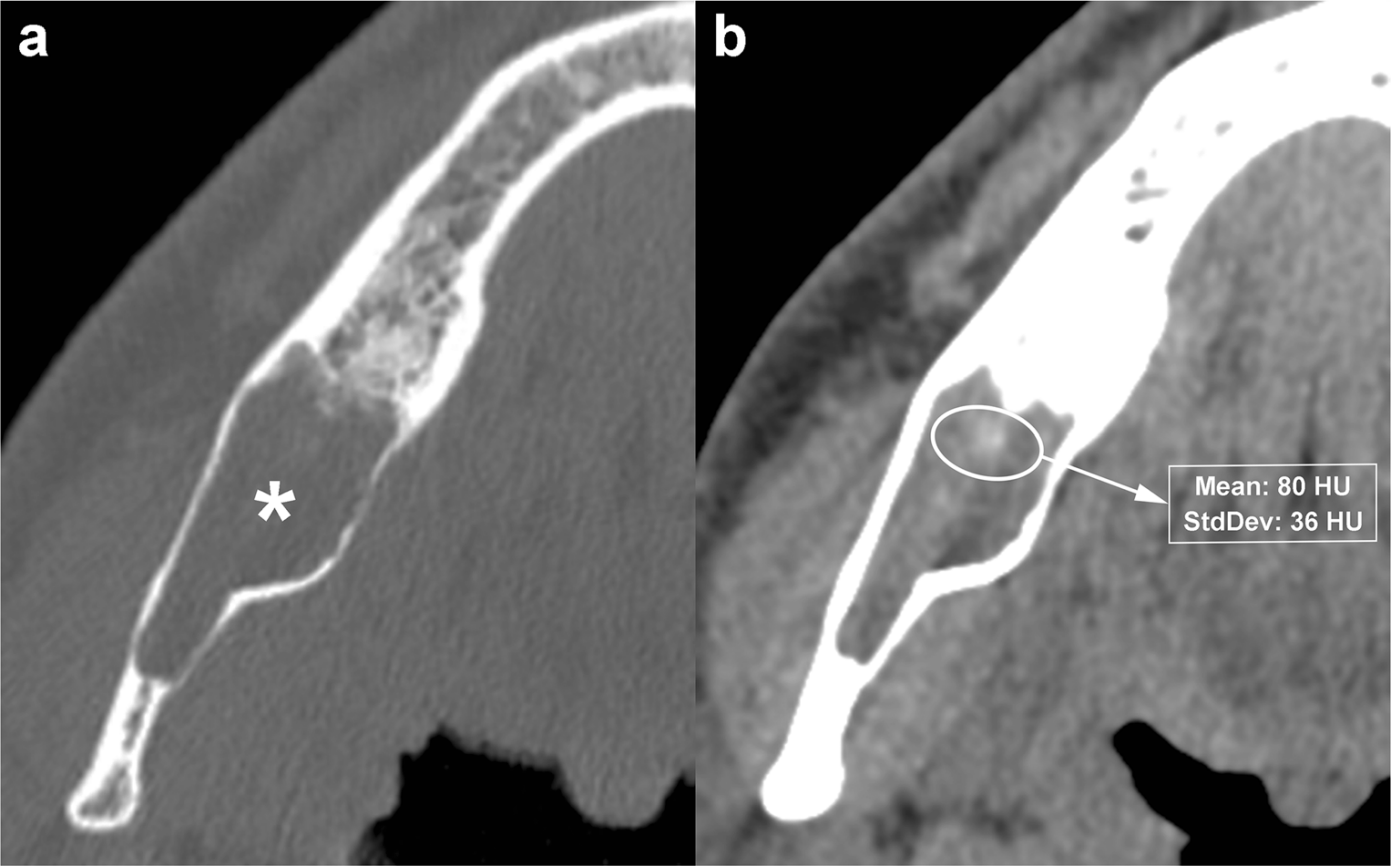
Axial MDCT image with bone window (**a**) shows an OKC in the posterior region of the right mandible (*asterisk*). Axial MDCT image with soft tissue window (**b**) clearly demonstrates a high-density area within the mandibular lesion (*ellipse ROI*) with a mean attenuation value of 80 HU

**Fig. 13:**
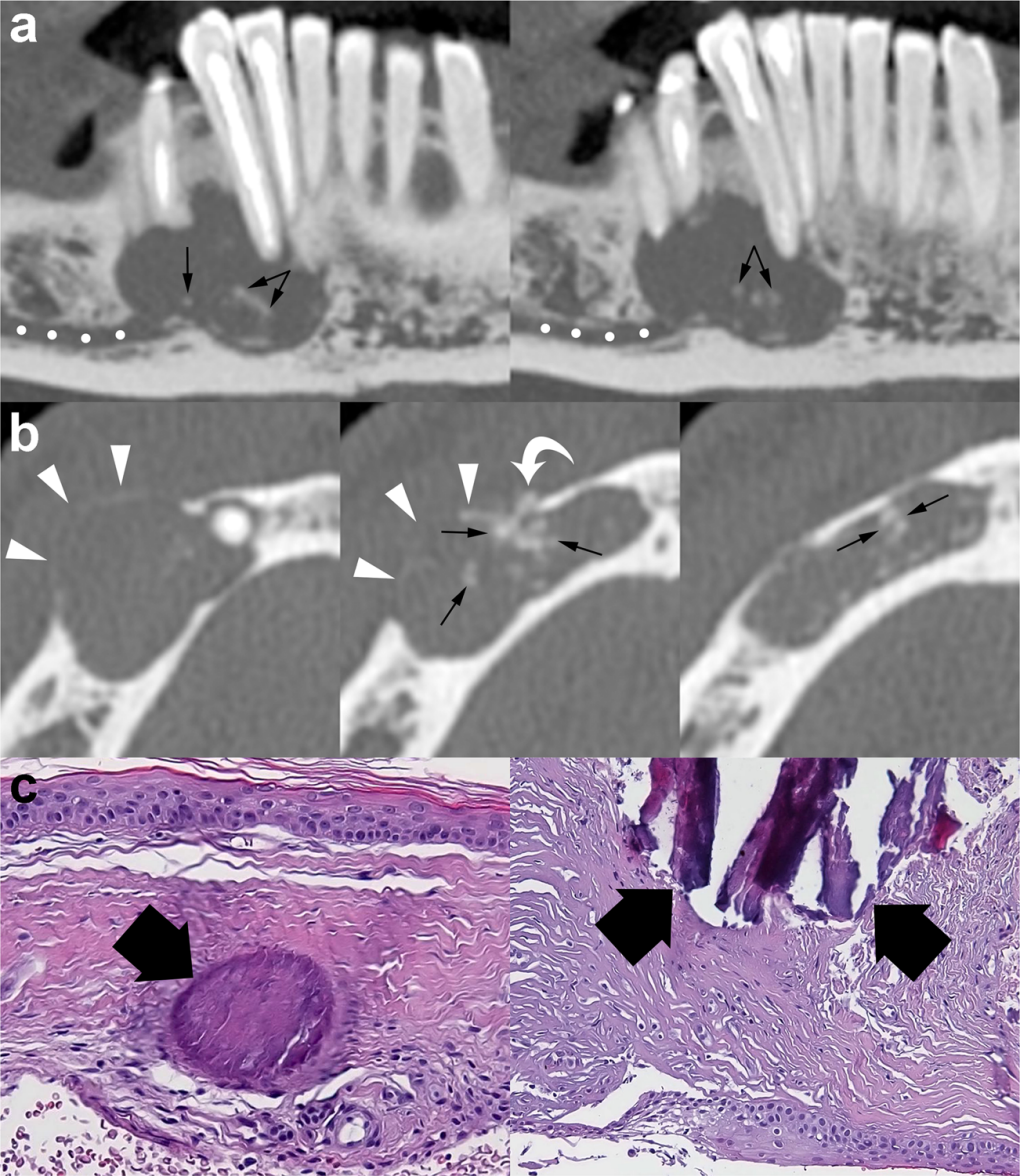
Panoramic (**a**) and axial (**b**) MDCT images show an osteolytic lesion located in the interforaminal region of the mandible. The lesion, histologically proven to represent an OKC, causes expansion and thinning of the buccal cortex (*arrowheads*). MDCT images demonstrate numerous punctate high-density foci (calcification) within the lesion (*arrows*). One of these high-density foci shows extension into adjacent soft tissue (*curved arrow*). *Dots*, mesial portion of the mandibular canal. **c** Histological images show the characteristic epithelial lining and calcifications (*large black arrows*) within the underlying connective tissue (H-E 10×)

Internal high-density areas are frequently found and reflect the presence of keratinised material within the OKC (Fig. 12) [14]. This peculiar internal feature is detectable mainly on MDCT scan due to its better soft tissue contrast discrimination compared to CBCT scan (Fig. 12). Although rare, calcifications may occur within OKCs; this finding is mostly observed in histological examinations (Fig. 13). Finally, at MDCT, the OKCs typically do not show enhancement after contrast administration [32].

### Magnetic resonance imaging

**Fig. 14:**
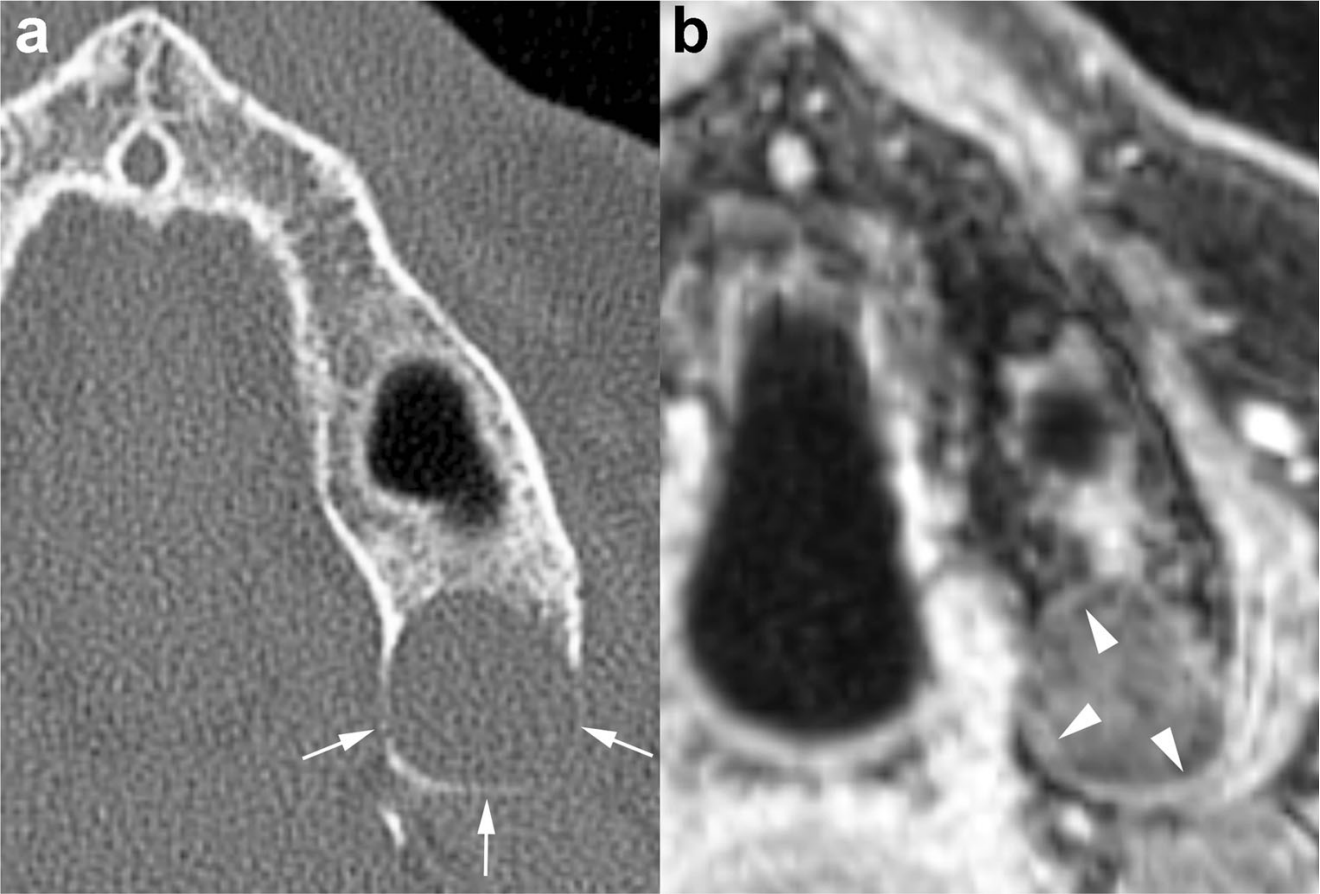
Unilocular OKC in the left maxillary tuberosity. **a** Axial MDCT image with bone window demonstrates remodelling and thinning of the adjacent cortices (*arrows*). **b** Note thin rim enhancement within the lesion on enhanced T1-weighted fat-saturated sequence (*arrowheads*)

**Fig. 15:**
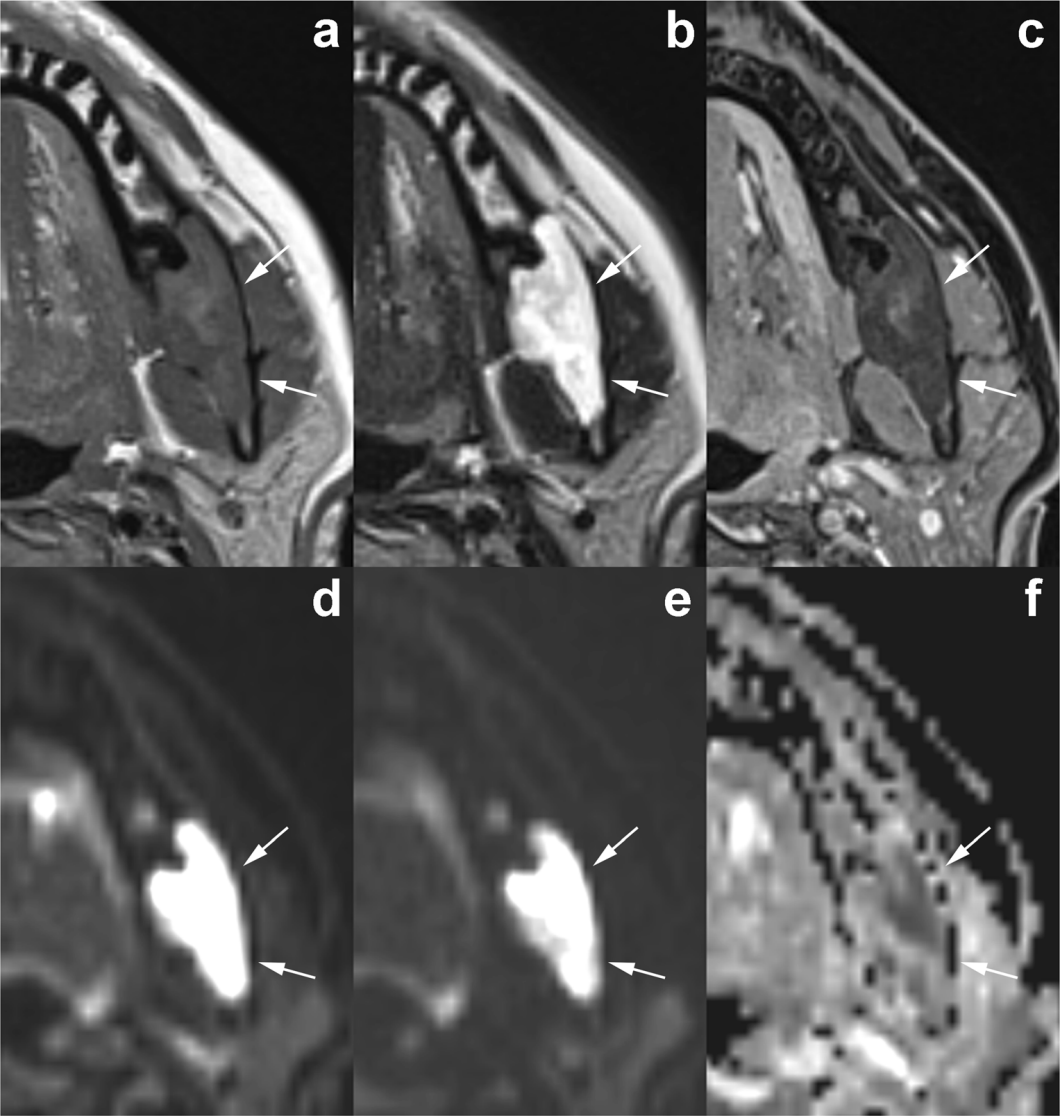
Axial magnetic resonance imaging (MRI) images demonstrate the typical signal pattern of OKC. The lesion, located in the posterior left mandible (*arrows*), shows intermediate-high signal intensity on T1-weighted sequence (**a**) and heterogenous high signal intensity on T2-weighted sequence (**b**). No enhancement is observed within the lesion on enhanced T1-weighted fat-saturated sequence (**c**). Diffusion-weighted imaging demonstrates restricted diffusion with high signal on b0 (**d**) and b1000 (**e**) images and low signal intensity on apparent diffusion coefficient (ADC) map (**f**)

In the evaluation of cystic lesions of the jaws, MRI is mainly performed as a complementary technique to CT (CBCT or MDCT), and it may be useful in selected cases to provide a better demonstration of the internal features and soft tissue involvement (Figs. 14 and 15).

OKCs typically show various signal intensity on MRI images, which reflect the materials contained inside the lesions. They are represented by a large amount of keratin sometimes associated with hyaline bodies in the presence of inflammation [34].

Various authors reported that most of the OKCs present intermediate or high signal intensity on T1-weighted sequences and heterogeneous signal intensity (from low to high) on T2-weighted sequences (Fig. 15) [20, 32, 34, 35]. Some studies have outlined that these MRI signal features are useful in discriminating between OKCs and ameloblastomas [34–37]. In a retrospective study including 19 ameloblastomas and 14 OKCs, Fujita et al. [37] compared signal intensity uniformity values of the cystic components of the two types of odontogenic lesions. In agreement with other authors, they observed that the cystic components of ameloblastomas and OKCs displayed significantly different uniformity values on all sequences. In particular, both unicystic and multicystic ameloblastomas show a more homogeneous signal intensity that is low on the T1-weighted images and high on the T2-weighted images [35, 36]. Moreover, cystic ameloblastomas typically have a thick and irregular enhancing wall, with or without papillary projections or intralesional nodules [36]. On the other hand, OKCs tend to be associated with thin and regular rim-enhancement on T1-weighted images (Fig. 14) [10, 36].

MRI with diffusion-weighted imaging (DWI) and calculation of apparent diffusion coefficient (ADC) is sensitive to physiological parameters such as tissue cellularity, nucleus-to-cytoplasm ratio and integrity of cell membranes, thus providing information about the microstructure of living tissues [38]. DWI may be useful as an adjunct tool for differentiation between OKCs and other odontogenic tumours, which may have overlapping imaging findings on conventional MRI sequences [39, 40].

In particular, as first demonstrated by Sumi et al. [39], the ADC value of OKCs is usually significantly lower than that of cystic/predominantly cystic ameloblastomas (Fig. 15). In a study by Srinivasan et al., the mean ADC value of OKCs was 1.019 ± 0.07 × 10^− 3^ mm^2^ s^− 1^ and the optimum cut-off for the differentiation with predominantly cystic ameloblastomas was 2.013 × 10^− 3^ mm^2^ s^− 1^ [40]. These findings reflect the higher viscosity of the content of OKCs determined by the presence of floating desquamated keratin, while the cystic spaces of ameloblastomas usually contain slightly proteinaceous fluids, sometimes with colloidal materials [39]. On the other hand, benign odontogenic cysts may present a wide range of ADC values due to the varying degrees of inflammatory cells infiltration [41].

Sakamoto et al., in a retrospective study including 35 odontogenic cystic lesions, showed that diffusion kurtosis imaging (DKI) could represent a quantitative evaluation tool for better differentiating OKCs from other cystic lesions [41]. Indeed, DKI provides deeper information about tissue’s structural complexity and the combination of its parameters seems to have the potential to distinguish between simple fluid viscosity and the degree of restricted diffusion caused by floating substances and, as a consequence, to increase the diagnostic accuracy for differentiating between OKCs and odontogenic cysts, compared with ADC [41].

## Image interpretation keys and differential diagnosis

Radiological imaging, mainly CT (CBCT or MDCT) and, in selected cases, MRI, plays an important role in the diagnosis of OKCs. However, OKCs, in particular smaller lesions, may exhibit imaging features almost indistinguishable to other osteolytic jaw lesions. Hence, in order to obtain a definitive diagnosis, a histopathological examination is required [14]. From this point of view, radiological imaging is considered to be more useful in evaluating the extent and the effects on adjacent structures, rather than in characterising a lesion.

It is reported that, in some OKCs, the combination of clinical and radiological findings allows narrowing the differential diagnosis and, in some cases, making the correct diagnosis [18].

The imaging findings which are more effective for making a provisional diagnosis of OKC are:

- Well-defined unilocular osteolytic lesion in the posterior region of jaws (Fig. 10)
- Large osteolytic mandibular lesion with few septa and minimal buccolingual expansion (Fig. 6)

However, when an OKC is associated with an impacted tooth, it may simulate a dentigerous cyst. Similarly, when an OKC is multilocular and located in the posterior sextant or the ramus of the mandible, it may mimic an ameloblastoma. Finally, when an OKC has a periapical position or involves an edentulous area, it may be mistaken for a radicular cyst. As a result, dentigerous cyst, ameloblastoma and radicular cyst are considered the most common odontogenic lesions in the differential diagnosis of an OKC [32].

The imaging features which are more effective for suggesting a diagnosis of dentigerous cyst rather than of OKC are [14, 32]:

- Unilocular osteolytic lesion around the crown of impacted tooth
- No septa or loculation within the cyst
- More buccolingual expansion in mandible
- More homogeneous and high T2-weighted signal on MRI

The imaging features which are more effective for suggesting a diagnosis of ameloblastoma rather than of OKC are [14, 32]:

- Multilocular osteolytic lesion with multiple internal septa
- More buccolingual expansion in mandible
- More prominent tooth displacement and root resorption
- Post-contrast enhancement of septa and mural nodule (more easily detectable on MRI rather than on MDCT)
- Mean ADC value higher than 2.013 × 10^− 3^ mm^2^ s^− 1^ on DWI [40]

Finally, the imaging features which are more effective for suggesting a diagnosis of radicular cyst rather than of OKC are [14, 15, 32, 33]:

- Round or pear-shaped unilocular osteolytic lesion
- Epicentre at the apex of a non-vital tooth
- Iron-like density within the cyst (indicator of endodontic overfilling)

The typical features of OKCs, dentigerous cysts, radicular cysts and ameloblastomas are summarised in Table 1.

**Table 1:** Typical characteristics of odontogenic keratocysts (OKCs), dentigerous cysts, ameloblastomas and radicular cysts

Odontogenic lesion	Age (decade)	Gender	Predominant jaw	Predominant location	Image interpretation keys
Odontogenic keratocysts	3rd	M > F	Mandible	Posterior	Unilocular osteolytic lesion with few septa and growth along the length of the bone with minimal buccolingual expansion
Dentigerous cysts	2nd–3rd	M > F	Mandible	Posterior	Unilocular osteolytic lesion around the crown of impacted tooth with buccolingual expansion and no septa
Ameloblastomas	3rd–5th	M > F	Mandible	Posterior	Multilocular osteolytic lesion with thick septa, root resorption and buccolingual expansion
Radicular cysts	3rd–5th	M ~ F	None	None	Unilocular osteolytic lesion around the apex of a non-vital tooth

## Syndromic and non-syndromic multiple OKCs

The presence of multiple OKCs is considered one of the major criteria for the diagnosis of NBCCS, and their occurrence may be the first sign of the disease [42]. NBCCS, also known as Gorlin–Goltz syndrome, is an autosomal dominant multisystemic disease characterised by multiple nevoid basal cell carcinoma, multiple OKCs, palmar or plantar pits, calcifications of falx cerebri and skeletal abnormalities, such as bifid, fused or splayed ribs [43, 44].

Other features associated with NBCCS include craniofacial, neurological, sexual, ophthalmic and cardiac anomalies [45]. The literature reported that NBCCS is associated with mutations of a tumour suppressor gene, also called the PTCH gene. Mutations within the PTCH gene are also observed in some non-syndromic OKCs. Therefore, certain authors indicate that the abnormalities of the PTCH gene may be involved in the pathogenesis of OKCs [46].

Multiple OKCs are also observed in other syndromes, such as Noonan syndrome, Ehlers–Danlos syndrome and oral-facial-digital syndrome.

**Fig. 16:**
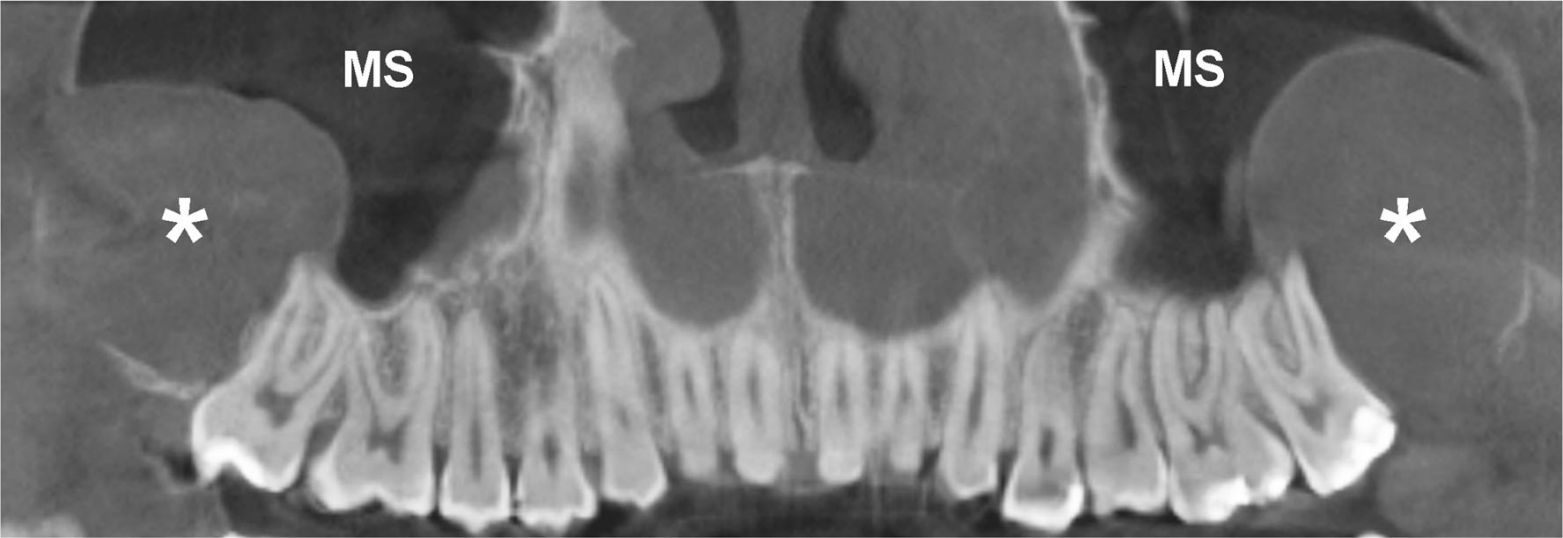
Panoramic CBCT image (1 mm thick) shows two unilocular OKCs on both sides of the maxilla (*asterisks*) in a young male patient with nevoid basal cell carcinoma syndrome (NBCCS). Both lesions, located in the posterior sextants, extend into the maxillary sinuses (*MS*)

In syndromic OKCs, the cysts occur at an early age (first or second decades of life), originate more often in the posterior sextants of the maxilla (Fig. 16), have more aggressive behaviour and their recurrence rate is higher than non-syndromic OKCs [44].

In rare case, multiple OKCs can be observed without any evidence of systemic disease [47].

However, it should be noted that the occurrence of multiple OKCs should indicate, until proven otherwise, the presence of a syndrome, and a patient with multiple OKCs should be followed regularly to assess the possible appearance of any other systemic manifestations.

## Treatment and follow-up

The management of OKCs aims to reduce the risk of recurrence while minimising, at the same time, the morbidity for the patient. At the present moment, there is no consensus about the best treatment modality.

Different factors take part in the choice of the more appropriate treatment, including size and location of the lesion, unilocularity or multilocularity, presence of cortical perforation or soft tissue involvement and the patient’s age.

Various surgical options have been considered, including enucleation alone or associated with adjunctive measures (ostectomy, Carnoy’s solution, cryotherapy), marsupialisation and decompression, marginal or segmental resection [19].

In a systematic review of the literature, Johnson et al. showed that enucleation is associated with the highest recurrence rate of about 30%, followed by marsupialisation alone (approximately 18% recurrence rate). The association of lesion’s enucleation with adjunctive technique of chemical cauterisation with Carnoy’s solution, a mixture of chloroform, absolute ethanol, glacial acetic acid and ferric chloride, significantly reduced the recurrence rates to about 8% [23].

Surgical resection, both marginal and segmental, is related to the lowest recurrence rate but, because of its morbidity, is not recommended as a primary treatment modality and should be reserved for retreatment of patients suffering from multiple recurring lesions [23].

According to the literature, most recurrences of OKC occur within the first 5–7 years after treatment [13].

**Fig. 17:**
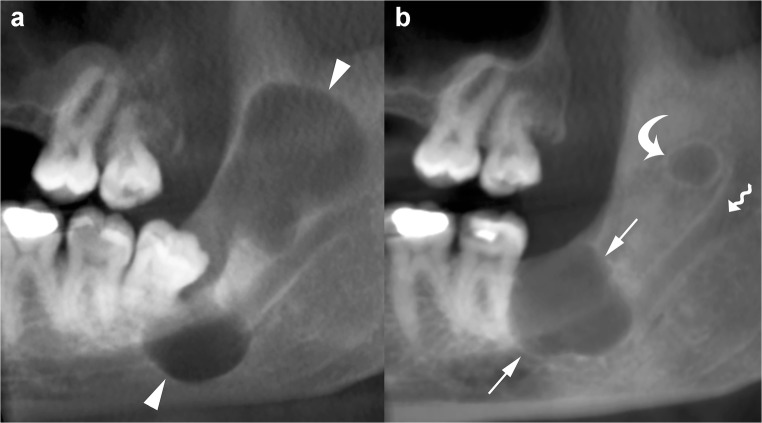
Panoramic CBCT images (20 mm thick) at baseline (**a**) and at the first follow-up (**b**). At baseline (**a**), a large unilocular OKC in the posterior mandible and ramus is shown (*arrowheads*). At the first follow-up (**b**), performed 2 years after surgery, the CBCT image shows two recurrences in the third molar region (*arrows*) and ramus of the mandible (*curved arrow*), respectively. *Wavy arrow*, mandibular foramen

In a paper by Apajalahti et al., the mean recurrence time was relatively shorter (about 2 years). This can be explained by the systematic use of CT in the follow-up of large OKCs (Fig. 17), thus helping the radiologist to depict very small lesions that not clinically detectable [48].

**Fig. 18:**
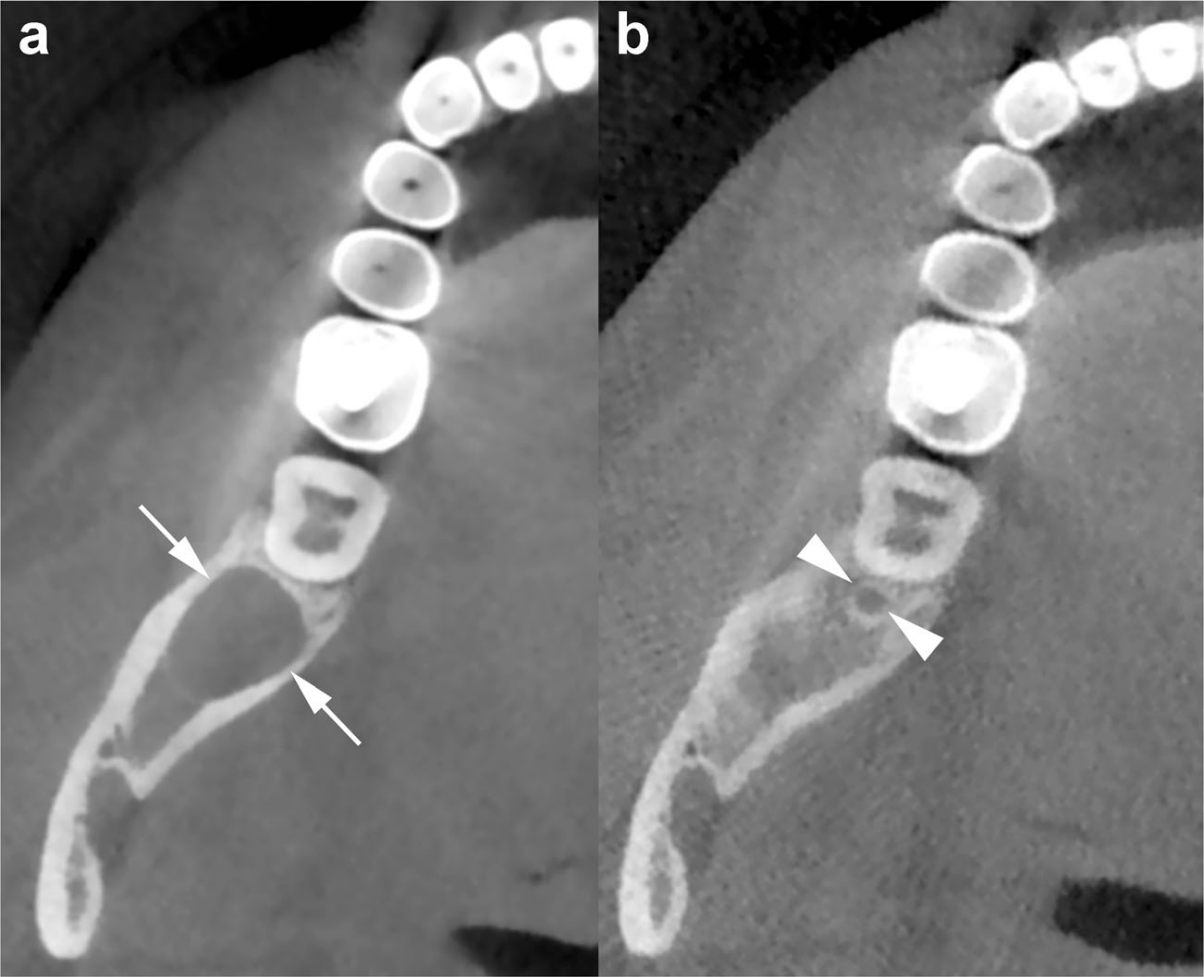
Periodic follow-up of the same patient in Fig. 16 with NBCCS. **a** Axial CBCT image shows a new lesion in the third molar region of the right mandible (*arrows*), which was subsequently surgically treated. **b** A postoperative axial CBCT image obtained 1 year later demonstrates a very small recurrence at the site of the previous lesion (*arrowheads*)

For this reason, periodic radiographic monitoring of patients with surgically treated OKCs is recommended annually for the first 5 years and at least every 2 or 3 years subsequently [20]. Patients with NBCCS are particularly prone to the formation of new lesions, both in the site of previous surgery and in different sextants of the dental arches (Fig. 18). Consequently, a long-term strict radiological follow-up should be performed in these patients [23].

## Conclusions

Odontogenic keratocysts (OKCs) are benign lesions of odontogenic origin accounting for about 10% of all odontogenic cysts and characterised by an aggressive behaviour.

Radiological imaging, mainly computed tomography (CT) and, in selected cases, magnetic resonance imaging (MRI), plays an important role in the diagnosis and management of OKCs. Although radiological imaging does not always provide a specific diagnosis, the knowledge about typical and atypical radiological features of OKCs is essential for their diagnosis and treatment planning. In particular, the combination of clinical and radiological findings is useful in evaluating the extent of the lesions and the relationships with adjacent structures.

The relatively high recurrence rate, especially after conservative surgery, make it necessary to perform a periodic radiographic monitoring of patients with surgically treated OKCs, at least for the first 5 years.

## Abbreviations

ADC: Apparent diffusion coefficient
CBCT: Cone beam computed tomography
CT: Computed tomography
DKI: Diffusion kurtosis imaging
DWI: Diffusion-weighted imaging
KCOT: Keratocystic odontogenic tumour
MDCT: Multidetector computed tomography
MPR: Multiplanar reconstruction
MRI: Magnetic resonance imaging
NBCCS: Nevoid basal cell carcinoma syndrome
OKC: Odontogenic keratocyst
PTCH: Protein patched homolog
WHO: World Health Organization
